# LAP-defined hepatic steatosis and cardiometabolic risk in rheumatoid arthritis: a post-hoc analysis of the FRANCIS randomized trial

**DOI:** 10.1007/s00296-026-06254-6

**Published:** 2026-07-24

**Authors:** A. N. Saidi, B. Burggraaf, A. J. van der Lelij, E. van der Zwan-van Beek, M. Castro Cabezas

**Affiliations:** 1 Department of Internal Medicine, Centre of Endocrinology, Diabetes and Vascular Medicine, Franciscus Gasthuis & Vlietland, Rotterdam, 3045 PM The Netherlands; 2 Department of Internal Medicine, Erasmus University Medical Center, Rotterdam, The Netherlands; 3 Department of Clinical Chemistry, Franciscus Gasthuis & Vlietland, Rotterdam, The Netherlands; 4 Julius Clinical, Zeist, The Netherlands

**Keywords:** Arthritis, rheumatoid, Fatty liver, Carotid intima-media thickness, Cardiovascular diseases, Atherosclerosis, Lipid accumulation product

## Abstract

Rheumatoid arthritis (RA) is a chronic autoimmune disease associated with metabolic dysfunction-associated steatotic liver disease (MASLD), which may contribute to cardiovascular (CV) risk. This study aimed to examine the association between hepatic steatosis, measured by a non-invasive test, and CV risk markers in RA patients. This post-hoc analysis used cross-sectional and longitudinal data from the Franciscus Rheumatoid Arthritis and Cardiovascular Intervention Study (FRANCIS), an open-label randomized controlled trial of intensive CV risk management in patients with rheumatoid arthritis without cardiovascular disease (CVD) or type 2 diabetes. Hepatic steatosis was assessed using the Lipid Accumulation Product (LAP) with sex-specific thresholds for rule-in and rule-out of severe steatosis. Associations between hepatic steatosis and carotid intima-media thickness (cIMT) were assessed using linear regression analysis. Crude associations were first evaluated in unadjusted models, followed by multivariable models adjusting for age, sex, BMI, and systolic blood pressure. Longitudinal changes in cIMT over 3 years were analyzed according to LAP-defined steatosis and treatment strategy. Among 236 RA patients (66% female; mean age 54 ± 10 years), those with LAP-defined steatosis had a more adverse cardiometabolic profile, including higher blood pressure, fasting glucose, and a more atherogenic lipid profile (all *p* < 0.05). Mean cIMT tended to be higher in the steatosis group (0.61 ± 0.10 vs. 0.57 ± 0.11 mm). In crude regression, LAP-defined steatosis showed a non-significant trend toward higher cIMT (B = 0.036, 95% CI − 0.003–0.076), which was no longer observed after multivariable adjustment (B = − 0.016, 95% CI − 0.050–0.019). Over 3 years, intensive treatment reduced cIMT progression compared with standard treatment in patients without LAP-defined steatosis (ΔcIMT 0.03 ± 0.07 vs. 0.06 ± 0.12 mm; *p* = 0.013), whereas no treatment effect was observed in patients with LAP-defined steatosis (ΔcIMT 0.05 ± 0.05 vs. 0.04 ± 0.05 mm; *p* = 0.696). Intensive treatment also produced greater improvements in lipid parameters, including reductions in total cholesterol and apoB in both groups, and increased apoAI mainly in participants with LAP-defined steatosis, while glycemic, hepatic, and inflammatory markers remained largely unchanged. In this exploratory post-hoc analysis, LAP-defined hepatic steatosis identified RA patients with a more adverse cardiometabolic profile. An unadjusted association with subclinical vascular burden was observed but was attenuated after adjustment for established CV risk factors. Intensive CV risk management was associated with lower cIMT progression in patients without LAP-defined steatosis, while no clear vascular effect was observed in those with LAP-defined steatosis. No evidence of differential treatment effects according to LAP-defined steatosis was found.

**Supplementary Information:**

The online version contains supplementary material available at 10.1007/s00296-026-06254-6.

## Introduction

Metabolic dysfunction-associated steatotic liver disease (MASLD), formerly known as non-alcoholic fatty liver disease (NAFLD) [1–3], affects approximately 30% of the general population and is characterized by excessive hepatic fat accumulation in the presence of at least one cardiometabolic risk factor, in the absence of secondary causes of hepatic steatosis, or excessive alcohol intake [4, 5]. While early-stage steatosis is reversible, progression to liver fibrosis is a key determinant of MASLD prognosis and is associated with increased morbidity and mortality [6–9].

MASLD is also common in patients with rheumatoid arthritis (RA), a chronic autoimmune disease with elevated cardiovascular (CV) risk [1]. Approximately one-third of RA patients are affected by MASLD [10, 11], which can range from simple steatosis to more severe forms such as metabolic dysfunction-associated steatohepatitis (MASH), potentially progressing to cirrhosis or hepatocellular carcinoma (HCC) [12]. Fibrosis, in particular, is an important CV risk factor, both in RA and non-RA populations, as demonstrated in our previous work [13].

RA is associated with increased cardiovascular disease (CVD) risk, driven by systemic inflammation and often undertreated traditional CV risk factors [6–9]. Patients with RA have a 1.5-fold higher risk of CVD and increased CVD-related morbidity and mortality compared with the general population [6, 8, 9, 14, 15]. Subclinical vascular damage is also evident, as carotid intima-media thickness (cIMT), a marker of atherosclerosis, is frequently elevated in RA patients [13, 16, 17].

Despite the close interconnection between MASLD and CVD and the role of inflammation in both conditions, it remains unclear whether hepatic steatosis contributes independently to CV risk in RA beyond established cardiometabolic risk factors. While liver biopsy remains the gold standard for assessing hepatic fat content and fibrosis, its invasive nature, potential complications, and high cost limit its routine use [18]. Non-invasive tests (NITs), such as the lipid accumulation product (LAP) and the Fibrosis-4 index (FIB-4), are widely used surrogate markers of hepatic steatosis and fibrosis, respectively, and are recommended by the European Association for the Study of the Liver (EASL) guidelines [4, 10, 11].

The present study therefore investigates the association between hepatic steatosis, assessed using LAP, and CV risk markers, including cIMT, in patients with RA, and evaluates whether longitudinal changes in cIMT differ according to treatment strategy and LAP-defined steatosis categories.

## Methods

### Study design

This is a post-hoc analysis of the Franciscus Rheumatoid Arthritis and Cardiovascular Intervention Study (FRANCIS), an open-label randomized controlled trial designed to assess the impact of intensive CV risk management in patients with RA [9, 19, 20]. Patients were randomized to receive either standard treatment or an intensive treat-to-target treatment regarding CV risk factors, as previously described [9]. In short, patients within the treat-to-target group were treated according to a pre-specified protocol for anti-hypertensive treatment, lipid lowering drugs, metformin, and life style advice including diet and smoking cessation. Patients with the standard treatment were only evaluated and their general physician received written advice based on results of the visits, however, initiation of treatment was based on their own judgement and CVD risk guidelines. The study protocol was approved by the Medical Ethical Committee of Maasstad Hospital (approval date: 20 October 2010; ABR no. NL32669.101.10), Rotterdam, and conducted in accordance with the Declaration of Helsinki [21]. The trial was registered with The Dutch Trial Register (NTR3873; ABR no. NL32669.101.10).

### Study population

The study cohort included 236 RA patients attending the outpatient clinic of the Department of Rheumatology at Franciscus Gasthuis Hospital, Rotterdam, the Netherlands. Eligible participants were younger than 70 years and had a confirmed diagnosis of RA according to the American College of Rheumatology criteria [22]. Patients with type 1 or type 2 diabetes mellitus at baseline - defined by fasting glucose > 7.0 mmol/L, HbA1c > 48 mmol/mol, or ongoing treatment for diabetes - were excluded. Individuals with a history of CVD, defined as prior myocardial infarction, cerebrovascular events, intermittent claudication, peripheral artery disease requiring amputation, coronary artery bypass grafting, or percutaneous coronary interventions, were also excluded. Additional exclusion criteria comprised chronic kidney disease, defined as an estimated glomerular filtration rate (eGFR) below 40 mL/min/1.73 m².

### Data collection

Baseline assessments were performed during outpatient visits between 2009 and 2012. Blood samples were collected following an overnight fast, and standard anthropometric measurements, including weight, height, and blood pressure, were recorded. Medication use was documented for disease modifying anti-rheumatic drugs (DMARDs) (including methotrexate, hydroxychloroquine, and leflunomide), biologicals (including rituximab and anti-TNF agents), steroids, non-steroidal anti-inflammatory drugs (NSAIDs), statins, and antihypertensives, RA disease activity was assessed using the 28-joint Disease Activity Score (DAS28) [23]. Longitudinal analyses of cIMT progression included patients with complete baseline and 3-year follow-up data for LAP and cIMT.

### Lipid accumulation product (LAP)

The natural logarithm of LAP (lnLAP) was computed using sex-specific formulas based on waist circumference (WC, cm) and fasting triglycerides (TGs, mmol/L):

$$\:\mathrm{L}\mathrm{A}\mathrm{P}\mathrm{m}\mathrm{e}\mathrm{n}\text{}=\left(\mathrm{W}\mathrm{C}-65\right)\times\:\mathrm{T}\mathrm{G}$$

$$\:\mathrm{L}\mathrm{A}\mathrm{P}\mathrm{w}\mathrm{o}\mathrm{m}\mathrm{e}\mathrm{n}\text{}=\left(\mathrm{W}\mathrm{C}-58\right)\times\:\mathrm{T}\mathrm{G}$$

Severe steatosis was defined using sex-specific thresholds of lnLAP > 4.0 for men and lnLAP > 4.4 for women, which were used to categorize patients as ‘rule-in’ or ‘rule-out’ for hepatic steatosis. LAP has been validated as a surrogate marker for hepatic fat content and metabolic risk in general and RA populations [24].

### Carotid intima media thickness (cIMT)

cIMT was assessed by trained sonographers using the ART-LAB ultrasound system (Esaote, Italy), following previously described protocols [19]. B-mode imaging was performed to capture the far wall of each common carotid artery, producing two echogenic lines corresponding to the intima and media layers. The distance between these lines was measured to determine the cIMT. Atherosclerotic plaque was defined as a localized thickening with a cIMT ≥ 1.0 mm.

### Laboratory measurements

For all participants, a standardized panel of laboratory tests was performed at the Department of Clinical Chemistry, Franciscus Gasthuis Hospital. Blood samples were collected following routine procedures. Liver enzymes, renal function markers, total cholesterol, HDL-C, glucose, C-reactive protein (CRP), and triglycerides (TGs) were analyzed using DxC analyzers or Synchron LX20 systems (Beckman Coulter, USA) [25, 26]. LDL-C concentrations were calculated using the Friedewald formula when TG levels were below 4.00 mmol/L.

### Data analysis

Data are presented as mean ± standard deviation (SD), unless otherwise specified. Normally distributed continuous variables were compared using independent-samples t-tests, while non-normally distributed variables were analyzed with Mann-Whitney U or Kruskal-Wallis tests. Differences in baseline characteristics across LAP-defined steatosis categories were evaluated using t-tests or ANOVA, with Levene’s test applied to assess equality of variances. Linear regression was used to assess the association between LAP-defined steatosis and cIMT, using crude and subsequently multivariable models adjusted for age, sex, BMI, and systolic blood pressure. Variance inflation factors (VIFs) were calculated to assess multicollinearity, with VIF values < 5 considered acceptable. Two-way ANOVA was used to test the interaction between treatment group and LAP-defined steatosis for longitudinal cIMT changes. Categorical variables were compared using chi-square tests. Changes in ΔcIMT from baseline to 3-year follow-up were analyzed using independent-samples t-tests, stratified by treatment group and LAP-defined steatosis. Statistical significance was defined as *p* < 0.05. Observations with missing data were excluded from the analyses. Analyses were performed using IBM SPSS Statistics version 28.0.0.0 (IBM, New York, USA).

## Results

### Baseline characteristics

A total of 236 patients with RA were assessed at baseline (66% female; mean age 54 ± 10 years; mean BMI 26.4 ± 4.6 kg/m²). Baseline characteristics according to LAP-defined hepatic steatosis (“rule-in” vs. “rule-out”) are summarized in Table 1. Median lnLAP in the total cohort was 3.6 (IQR 3.1–3.9), with 3.4 (3.0–3.8) in the rule-out group and 4.5 (4.3–4.9) in the rule-in group (*p* < 0.001). Baseline characteristics were generally comparable between treatment groups within each LAP-defined steatosis group (Supplementary Table S1). Patients with LAP-defined steatosis were older, had higher BMI and WC, and exhibited a more atherogenic lipid profile, including higher total cholesterol, TGs, apoB, and apoB48, and lower HDL-C and apoAI (all *p* < 0.05; see Table 1). They also had higher systolic blood pressure, and showed a non-significant trend toward higher diastolic pressure (*p* = 0.091), as well as higher fasting glucose compared with those without LAP-defined steatosis. Mean cIMT tended to be higher in the rule-in group (0.61 ± 0.10 mm vs. 0.57 ± 0.11 mm; *p* = 0.072). Antihypertensive therapy was more frequently used among patients with steatosis (35.3% vs. 15.4%; *p* = 0.006), whereas the use of statins, hydroxychloroquine, anti-TNF agents, prednisone, methotrexate, and NSAIDs did not differ between groups.

**Table 1:** Baseline characteristics of RA patients (*N* = 236) according to the Lipid Accumulation Product (LAP)

Variable	LAP rule-out (*N* = 202)	LAP rule-in (*N* = 34)	*P*-value
lnLAP	3.4 (3.0–3.8)	4.5 (4.3–4.9)	< 0.001
cIMT (mm)	0.57 ± 0.11	0.61 ± 0.10	0.072
Age (years)	54 ± 11	57 ± 7	0.024
Sex (f/m)	140(69.3)/62(30.7)	15(44.1)/19(55.9)	0.004
BMI (kg/m^2^)	25.9 ± 4	30.0 ± 5	< 0.001
WC (cm)	92 ± 11	109 ± 12	< 0.001
Total cholesterol (mmol/L)	5.4 ± 1.0	5.8 ± 1.0	0.027
HDL-C (mmol/L)	1.6 ± 0.4	1.2 ± 0.3	< 0.001
LDL-C (mmol/L)	3.4 ± 0.9	3.6 ± 0.9	0.133
TG (mmol/L)	0.98 (0.71–1.2)	2.0 (1.6–2.6)	< 0.001
ApoB (g/L)	0.98 ± 0.25	1.2 ± 0.26	< 0.001
ApoAI (g/L)	1.7 ± 0.4	1.5 ± 0.4	0.009
ApoB48 (mg/L)	8.0 (4.8–12)	14 (11–23)	< 0.001
Systolic BP (mmHg)	132 ± 18	139 ± 17	0.031
Diastolic BP (mmHg)	79 ± 10	82 ± 10	0.091
Fasting glucose (mmol/L)	5.4 ± 0.52	5.7 ± 0.48	0.032
HbA1c (mmol/mol)	35 ± 4.2	36 ± 5.4	0.473
CRP (mg/L)	3.0 (1.0–6.0)	2.0 (1.0–5.0)	0.303
ALT (U/L)	25 (18–31)	29 (23–37)	0.007
AST (U/L)	17 (13–21)	19 (15–22)	0.227
DAS28CRP	2.5 ± 1.1	2.3 ± 0.98	0.502
DAS28ESR	2.6 ± 1.2	2.3 ± 1.2	0.261
FIB-4	0.80 (0.54–1.1)	0.79 (0.67–1.1)	0.650
Antihypertensives (%)	31 (15.4)	12 (35.3)	0.006
Statins (%)	14 (7.0)	0 (0)	0.113
Hydroxychloroquine (%)	48 (23.8)	8 (23.5)	0.976
Anti-TNF (%)	71 (35.3)	12 (35.3)	0.997
Prednisone (%)	25 (12.4)	5 (14.7)	0.714
Methotrexate (%)	149 (74.5)	25 (73.5)	0.905
NSAID (%)	83 (41.3)	15 (44.1)	0.757

Data are expressed as mean ± standard deviation, median (IQR), or n (%), as appropriate. P-values indicate differences between “rule out” and “rule in” groups for each marker

*lnLAP* natural logarithm of the Lipid Accumulation Product (LAP), *cIMT* carotid intima media thickness, *BMI* body mass index, *WC* waist circumference, *HDL-C* high-density lipoprotein cholesterol, *LDL-C* low-density lipoprotein cholesterol, *TG* triglycerides, *ApoB* apolipoprotein B, *ApoAI* apolipoprotein AI, *ApoB48* apolipoprotein B48, *systolic BP* systolic blood pressure, *diastolic BP* diastolic blood pressure, *HbA1c* hemoglobin A1C, *ALT* Alanine Aminotransferase, *AST* Aspartate Aminotransferase, *DAS28CRP* Disease Activity Score using 28 joints with C-Reactive Protein, *DAS28ESR* Disease Activity Score using 28 joints with Erythrocyte Sedimentation Rate, *Anti-TNF* anti-tumor necrosis factor, *NSAID* non-steroidal anti-inflammatory drug.

* P-values derived from independent-samples t-test for normally distributed variables, Mann–Whitney U test for non-normally distributed variables, and chi-square test for categorical variables. Observations with missing values for medication variables (1–2 per variable) were excluded from the calculation of percentages and chi-square analyses.

### Association between LAP-defined steatosis and cIMT

In the crude linear regression, LAP-defined steatosis showed a non-significant trend toward higher cIMT (B = 0.036, 95% CI -0.003 to 0.076). After adjustment for age, sex, BMI, and systolic blood pressure, the association was no longer observed (B = -0.016, 95% CI -0.050 to 0.019) (Supplementary Table S2). Regression diagnostics showed no major deviations from model assumptions, and no relevant multicollinearity was observed among included covariates (all VIFs < 5).

In sensitivity analyses, LAP was compared with its individual components (WC and TGs). In the components model, neither WC nor TGs was independently associated with cIMT (*p* = 0.627 and *p* = 0.596, respectively). Similarly, in the model including LAP, LAP was not independently associated with cIMT (*p* = 0.447).

### Pre- and post-intervention changes in biochemical parameters

Pre- and post-intervention biochemical outcomes at 3 years are summarized in Table 2, stratified by baseline steatosis status and treatment group. Among patients without LAP-defined steatosis, body weight increased slightly in the intensive treatment group (75 ± 14 to 76 ± 14 kg, *p* = 0.033) but remained unchanged in the standard treatment group (*p* = 0.246). No significant weight changes were observed in participants with LAP-defined steatosis. Total cholesterol decreased in participants without LAP-defined steatosis in both treatment groups (5.3 ± 1.1 to 4.7 ± 1.0 mmol/L in the intensive group, *p* < 0.001; 5.5 ± 1.0 to 5.1 ± 0.8 mmol/L in the standard group, *p* < 0.001). In participants with LAP-defined steatosis, a significant reduction was observed only in the intensive treatment group (5.7 ± 1.2 to 4.7 ± 1.1 mmol/L, *p* = 0.002), whereas no significant change occurred with standard treatment (*p* = 0.131). HDL-C increased across treatment groups and steatosis categories (all *p* ≤ 0.005), and LDL-C decreased significantly in all groups (all *p* ≤ 0.035). TGs increased significantly in the standard treatment group among participants without LAP-defined steatosis (*p* < 0.001), while remaining stable with intensive treatment (*p* = 0.185), leading to a significant between-group difference (*p* = 0.002). ApoB decreased significantly with intensive treatment in both steatosis categories, whereas standard treatment led to a small but statistically significant increase among participants without LAP-defined steatosis, resulting in a significant between-group difference. ApoAI increased significantly only in participants with LAP-defined steatosis receiving intensive treatment (*p* = 0.041), while remaining largely unchanged in other groups. Fasting glucose and HbA1c increased modestly, primarily in participants without LAP-defined steatosis, while remaining stable in those with LAP-defined steatosis, with values remaining within the non-diabetic range. Inflammatory and hepatic markers (CRP, ALT, AST) did not change significantly in any group.

**Table 2:** Pre- and post-intervention changes in blood markers by steatosis category and treatment strategy

Variable	Treatment group	Rule-out steatosis (*N* = 202)				Rule-in steatosis (*N* = 34)			
		Baseline	Follow-up	N	P-value	Baseline	Follow-up	N	P-value
Weight (kg)	Standard	75 ± 13	76 ± 16	103	0.246	91 ± 18	89 ± 16	16	0.430
	Intensive	75 ± 14	76 ± 14	99	0.033	91 ± 16	92 ± 20	17	0.504
Total cholesterol (mmol/L)	Standard	5.5 ± 1.0	5.1 ± 0.8	103	< 0.001	6.0 ± 0.9	5.4 ± 0.9	16	0.131
	Intensive	5.3 ± 1.1	4.7 ± 1.0	97	< 0.001**	5.7 ± 1.2	4.7 ± 1.1	17	0.002
HDL-C (mmol/L)	Standard	1.5 ± 0.4	1.7 ± 0.4	103	< 0.001	1.2 ± 0.3	1.4 ± 0.3	16	0.005
	Intensive	1.6 ± 0.4	1.7 ± 0.4	97	< 0.001	1.1 ± 0.3	1.4 ± 0.4	17	< 0.001
LDL-C (mmol/L)	Standard	3.4 ± 0.9	2.9 ± 0.8	103	< 0.001	3.8 ± 0.7	3.0 ± 0.7	13	0.035
	Intensive	3.3 ± 1.0	2.5 ± 0.7	96	< 0.001*	3.5 ± 1.0	2.5 ± 0.6	16	< 0.001
Triglycerides (mmol/L)	Standard	1.1 (0.7–1.3)	1.2 (0.9–1.8)	103	< 0.001	1.7 (1.6–3.3)	1.7 (1.2–2.3)	16	0.088
	Intensive	0.9 (0.7–1.2)	1.0 (0.7–1.4)	97	0.185**	2.3 (1.8–2.7)	2.0 (1.5–2.6)	17	0.381
ApoB (g/L)	Standard	0.99 ± 0.3	1.0 ± 0.3	103	0.024	1.2 ± 0.3	1.2 ± 0.3	16	0.540
	Intensive	0.97 ± 0.3	0.90 ± 0.3	97	0.012**	1.2 ± 0.3	1.0 ± 0.2	17	0.025
ApoAI (g/L)	Standard	1.7 ± 0.4	1.7 ± 0.4	103	0.091	1.5 ± 0.3	1.5 ± 0.2	16	0.928
	Intensive	1.7 ± 0.4	1.7 ± 0.3	97	0.854	1.6 ± 0.4	1.7 ± 0.5	17	0.041
Fasting glucose (mmol/L)	Standard	5.4 ± 0.5	5.9 ± 1.4	102	< 0.001	5.7 ± 0.6	5.8 ± 0.7	17	0.715
	Intensive	5.5 ± 0.6	5.7 ± 1.4	98	0.092	5.6 ± 0.3	5.9 ± 0.9	17	0.147
HbA1c (mmol/mol)	Standard	35 ± 4.2	37 ± 4.1	101	< 0.001	36 ± 5.3	37 ± 4.3	17	0.337
	Intensive	35 ± 4.2	37 ± 5.4	97	< 0.001	36 ± 5.6	37 ± 5.0	17	0.289
CRP (mg/L)	Standard	3.0 (1.0–6.0)	2.0 (1.0–5.0)	103	0.380	1.0 (1.0–4.0)	1.0 (1.0–4.3)	17	0.373
	Intensive	3.0 (1.0–6.0)	2.0 (1.0–5.0)	99	0.519	3.0 (1.0–6.0)	2.0 (1.0–5.0)	17	0.058
ALT (U/L)	Standard	24 (18–31)	24 (18–30)	102	0.176	31 (26–38)	33 (23–41)	16	0.877
	Intensive	25 (19–32)	24 (16–30)	98	0.263	28 (21–36)	26 (19–34)	17	0.636
AST (U/L)	Standard	18 (13–21)	17 (13–22)	103	0.256	20 (17–28)	22 (16–26)	17	0.977
	Intensive	17 (13–21)	16 (13–23)	99	0.703	18 (13–21)	16 (13–27)	17	0.635

*HDL-C* high density cholesterol; *LDL-C* low density cholestero, *ApoB* apolipoprotein B, *ApoAI* apolipoprotein AI, *HbA1c* glycated hemoglobin, *CRP* C-reactive protein, *ALT* Alanine Aminotransferase, *AST* Aspartate Aminotransferase.

*Notes:* Total N per steatosis category (rule-out 202, rule-in 34); Ns for individual variables may be lower due to missing data. Data are presented as mean ± SD for normally distributed variables and median (IQR) for non-normally distributed variables. P-values reflect within-group changes from baseline to follow-up. *p < 0.05 and **p < 0.01 indicate differences in change between standard and intensive treatment.

### cIMT progression by treatment and LAP category

**Fig. 1:**
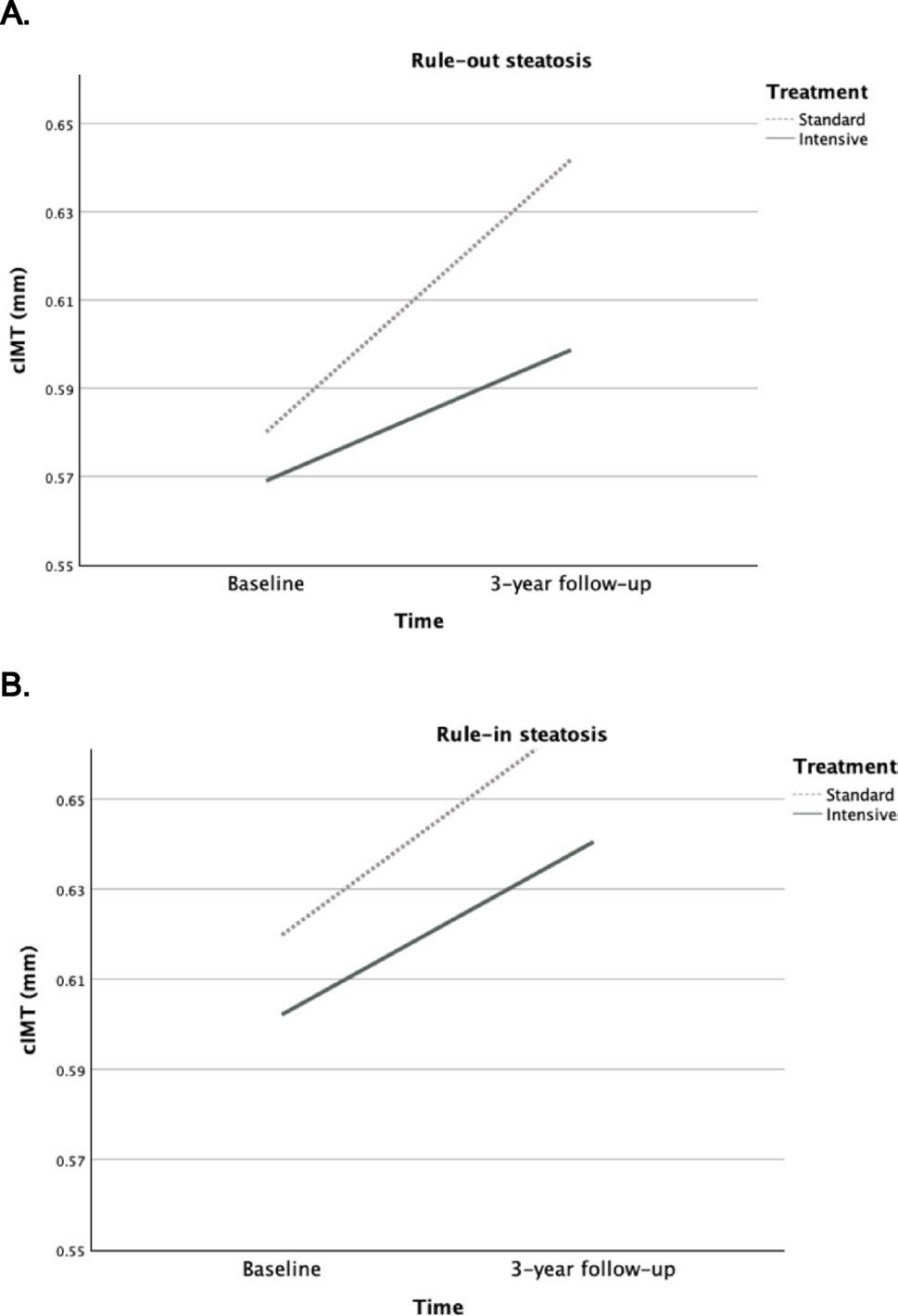
Mean cIMT over time in (**A**) patients without LAP-defined steatosis, and (**B**) patients with LAP-defined steatosis. Note: A significant difference in cIMT progression was observed between treatment groups in patients who were ruled out for steatosis (*p* = 0.013), whereas no significant difference was found in patients who were ruled in for severe steatosis (*p* = 0.696). The y-axis lower bound was set at 0.55 mm to improve visualization of cIMT variability

Changes in cIMT over 3 years varied according to LAP-defined steatosis and treatment strategy (Table 3; Fig. 1). In patients without LAP-defined steatosis, cIMT progression was lower in the intensive treatment group compared with the standard group (0.03 ± 0.07 mm vs. 0.06 ± 0.12 mm; *p* = 0.013). In patients with LAP-defined steatosis, cIMT progression did not differ between treatment groups (0.05 ± 0.05 mm vs. 0.04 ± 0.05 mm; *p* = 0.696). No statistically significant treatment-by-steatosis interaction was observed (*p* = 0.192).

**Table 3:** Carotid intima-media thickness (cIMT) progression over 3 years by treatment strategy and baseline lnLAP category

Baseline lnLAP category	Treatment group	*N*	ΔcIMT	*P*-value
Rule-out steatosis	Standard	103	0.06 ± 0.12	0.013
	Intensive	99	0.03 ± 0.07	
Rule-in steatosis	Standard	17	0.04 ± 0.05	0.696
	Intensive	17	0.05 ± 0.05	

*Notes:* ΔcIMT = change in carotid intima-media thickness from baseline to 3-year follow-up (mm); values are mean ± SD. Steatosis categories were based on baseline lnLAP. P-values represent post-hoc simple effects from the two-way ANOVA (treatment × lnLAP category) comparing standard versus intensive treatment within each category. No significant interaction was observed

## Discussion

In this exploratory study, LAP-defined hepatic steatosis identified patients with RA who exhibited an adverse cardiometabolic profile, including higher levels of classical CV risk factors. In unadjusted analyses, LAP-defined steatosis was also associated with higher baseline cIMT. However, these associations were attenuated after adjustment for age, sex, BMI, and blood pressure, indicating that the observed vascular differences are largely explained by established cardiometabolic risk factors rather than LAP-defined steatosis itself. Sensitivity analyses comparing LAP with its individual components yielded consistent results and similarly did not support an independent association with cIMT. Given the limited size of the LAP-defined steatosis subgroup, these findings should be interpreted with caution, as limited statistical power and the possibility of type II error cannot be excluded. Over three years, intensive CV risk management was associated with lower cIMT progression in patients without LAP-defined steatosis. In contrast, patients with LAP-defined steatosis showed improvements in metabolic parameters, particularly lipid-related measures, but no statistically significant differences in vascular outcomes between treatment strategies. Importantly, no statistically significant interaction between treatment strategy and LAP-defined steatosis was observed, suggesting no differential treatment response. However, subgroup analyses remain exploratory, and uncertainty due to small sample size precludes firm conclusions regarding differential treatment effects. Glycemic, hepatic, and inflammatory, and RA disease activity measures were comparable between LAP-defined steatosis groups. Accordingly, our data do not support an interaction between RA-related inflammatory burden and LAP-defined steatosis in relation to vascular outcomes. The absence of significant differences in vascular outcomes among patients with LAP-defined steatosis may therefore reflect limited statistical power rather than absence of a true effect.

Within our cohort, intensive CV risk management was associated with lower cIMT progression compared with standard care. This effect was most evident in patients without LAP-defined steatosis, whereas in patients with LAP-defined steatosis, improvements in lipid-related parameters were observed without corresponding differences in vascular outcomes. However, these metabolic changes did not translate into detectable differences in vascular structure over follow-up. As cIMT is a surrogate endpoint, these findings should be interpreted accordingly. Overall, vascular changes across groups appear to reflect underlying cardiometabolic risk burden, consistent with the absence of an independent association between LAP-defined steatosis and cIMT after adjustment. Residual confounding cannot be excluded, particularly given the observational nature of subgroup analyses and limited sample size.

In contrast to our findings, a sub analysis of the CLEAR Outcomes trial reported that hepatic steatosis was independently associated with increased CV risk, and that LDL-cholesterol lowering with bempedoic acid reduced events in high-risk individuals [27]. These discrepancies may reflect differences in study populations, sample size, and the use of hard clinical endpoints versus surrogate measures of subclinical atherosclerosis.

Our findings are in line with previous evidence in RA showing that metabolic liver disease is closely linked to IR and cardiometabolic dysfunction [28, 29]. In the general population, MASLD has been consistently associated with increased CV risk, with stronger associations observed in more advanced disease stages, particularly in the presence of fibrosis [30–33]. Prior work from our group similarly demonstrated that in RA liver fibrosis, rather than isolated steatosis, is more strongly associated with subclinical atherosclerosis [13].

Together, these observations suggest that metabolic liver abnormalities may primarily reflect systemic cardiometabolic burden in earlier stages, whereas more advanced stages of MASLD may be more closely linked to vascular injury and CV risk. Consistent observations have been reported in non-RA populations, where associations with CV outcomes are stronger in more advanced stages of MASLD [30–33].

While previous studies in RA have largely focused on hepatic steatosis in relation to disease activity [34] and drug-induced liver injury [35, 36], less is known about metabolic liver phenotypes in relation to longitudinal vascular outcomes, particularly within randomized cardiovascular risk intervention settings. To our knowledge, this is among the first studies to assess LAP-defined steatosis in relation to longitudinal vascular outcomes in RA, and to evaluate its contribution beyond established cardiometabolic risk factors.

Strengths of this study include the well-characterized RA cohort, longitudinal vascular assessment, and standardized CV evaluation within a randomized trial framework. Limitations should also be acknowledged. First, this was a post-hoc exploratory analysis not powered for steatosis-defined subgroup comparisons. Second, hepatic steatosis was assessed using LAP rather than imaging or histology, as no liver imaging or histological data were available. Although LAP is a validated surrogate marker of hepatic steatosis, it does not directly measure hepatic fat content and also reflects visceral adiposity and IR, which may limit the precision of steatosis classification. Third, residual confounding by RA-related disease characteristics and treatment exposure cannot be excluded. Fourth, the relatively small number of patients with LAP-defined steatosis likely limited power to detect subgroup-specific associations and treatment effects. Finally, the single-center design may limit generalizability.

From a clinical perspective, LAP may serve as a simple and inexpensive tool to identify RA patients with an adverse cardiometabolic profile, but it does not appear to provide independent information on subclinical vascular disease beyond established risk factors. Future studies using imaging-based assessment of hepatic steatosis in larger RA cohorts are needed to clarify the relationship between metabolic liver phenotypes and CV risk, and to determine whether these markers provide additional information beyond established cardiometabolic risk factors.

## Conclusion

In conclusion, in RA, LAP-defined hepatic steatosis identifies patients with an adverse cardiometabolic profile but is not independently associated with baseline cIMT after adjustment for traditional CV risk factors. These findings suggest that the observed associations are largely explained by underlying metabolic risk rather than steatosis per se. Over follow-up, intensive CV risk management was associated with lower cIMT progression in patients without LAP-defined steatosis, whereas patients with LAP-defined steatosis showed improvements in metabolic parameters without statistically significant differences in vascular outcomes. No statistically significant differences in treatment effects between groups were observed. Given the post-hoc design and limited subgroup size, these findings should be interpreted as exploratory. Larger prospective studies are needed to clarify the relationship between metabolic liver phenotypes and CV risk in RA and to evaluate their potential clinical utility beyond established cardiometabolic risk factors.

## Supplementary Information

Below is the link to the electronic supplementary material.

Supplementary Material 1

## Data Availability

The datasets generated during and/or analyzed during the current study are available from the corresponding author on reasonable request.

## Declarations

### Conference presentation

No part of this work has been previously presented at a conference or published as a congress abstract.
